# Digital divide in nursing education: An issue of social justice

**DOI:** 10.4102/hsag.v28i0.2513

**Published:** 2023-10-31

**Authors:** Agnes Makhene

**Affiliations:** 1 Department of Nursing, Faculty of Health Sciences, University of Johannesburg, Johannesburg, South Africa

The advent of COVID-19 came with teaching and learning challenges for nurse educators. A world of technology opened up for use in teaching and learning. New technologies such as virtual reality, telemedicine and telehealth among others, emerged for both the classroom and clinical area. This has presented new opportunities as well as challenges for nursing education. The pedagogical and didactic demands should not be overlooked as we embark on this journey of technologies in teaching and learning. The digital divide, a social justice issue remains a reality for the South African nursing education fraternity. The pedagogical demand is that students should be the centre of teaching and learning; however, the past 3 years have exposed the digital divide that exists among society in the country but more so in nursing education. According to Maceviciute and Wilson (2018), digital inequality or unequal diffusion and adoption of digital goods and services are usually based on economic, social, geographical and generational divides. On the other hand, the American Nurses Association (ANA 2015) defines social justice as ‘the analysis, critique, and change of social structures, policies, laws, customs, power, and privilege that disadvantage or harm vulnerable social groups through marginalisation, exclusion, exploitation, and voicelessness’.

Cronenwett et al. (2007) in their article on quality and safety nursing education cited the use information and technology to communicate, manage knowledge, mitigate error, and support decision-making as an important aspect in educating nurses. The question remains, how do we realise this when digital divide still remains?

Literature has shown that the gap in access to information and communication technologies (ICTs) between the haves (the privileged class) and the have-nots (the underprivileged class) is rather wide (Venkatesh & Sykes 2013). Innovations like telehealth which includes the use of electronic information and telecommunications technologies to support and promote long-distance clinical health care, patient and professional health-related education, public health and health administration has further increased the digital divide in nursing practice and education (ANA 2021). This is true in nursing education. To learn adequately and efficiently in today’s technological arena nursing students need high-speed Internet connection, also known as broadband, which is an essential educational infrastructure in the 21st century. Those without access or adoption are in the digital divide. This structural reality as described by Neidig (2017) and Gilliard (2016) effectively translates to what is referred to as ‘digital redlining’ and further perpetuates social and economic disparities in nursing education. 21st century educational trends are marked by scientific and technological integration, allowing for academic participation, and monitoring of all who are involved, using the support of ICTs. These development strategies and academic perspectives are fundamentally generated by digital platforms, technologies and methods based on an educational scenario with new conditions for self-learning mediated by virtual environments (Lira et al. 2020). The inequalities in nursing education have since been glaringly exposed. The post-pandemic era has brought to attention how wide the lines of inequalities are among nursing students. The digital divide gap was widened by the demand to change to online teaching and learning modalities in higher education in the latter part of 2019–2022. This opened the nurse educator to socially unjust practices because we teach diverse groups of students with diverse economic, educational and techno literacy backgrounds. On the other hand, the scholarship of teaching and learning (SoTL) purports that teaching and learning should include a critical and transformative or social justice orientation, a phenomenon not yet addressed in nursing education (Kreber 2013a).

In addition Kreber (2013b) alludes to the need of scientific inquiry into what should be done to enhance scholarship of teaching and learning (SoTL) which involves the innovative integration of technology and artificial intelligence (IR) in nursing education in this era of the 4th industrial revolution (4th IR). This is a reality of an ideal world of teaching and learning that will not be realised if issues of justice and equality in and through higher education are not addressed.

Access to the Internet and Wi-Fi and ability to engage with digital literacies are important aspects of participatory parity and social justice in higher education, for both the students and nurse educators. Failure to address such challenges and disparities constitutes social injustice in nursing education (Leibowitz & Bozalek 2016). Furthermore, Leibowitz and Bozalek (2016) argue that the transformative perspective includes those structural injustices pertaining to international or global issues such as the digital divide and differential access to knowledge production and consumption, which all impact groups of people across national territories and individual higher educational or disciplinary contexts, including nursing education.

Nurse educators are called upon to become change agents by working purposefully and intentionally with other stakeholders in nursing education to challenge the status quo and develop socially just nursing education and inclusion. Pantić and Florian (2015) cite possibilities of combining theories of inclusive pedagogy and teacher agency for developing educators as agents of inclusion and social justice in nursing education. These possibilities include: nurturing commitment to social justice as part of the nurse educator’s sense of purpose; developing competencies in inclusive pedagogical approaches, including working with others; developing relational agency for transforming the conditions of nurse educator’s workplaces and a capacity to reflect on their own practices and environments when seeking to support the learning of all students. Nurse educators need to understand how broader social forces such as digital divide influence exclusion and disadvantage (Pantić & Florian 2015). Historical trends in data pursuant to structural inequities impacting disproportionally black people and other racialised minorities demonstrated that there may in fact be a digital and technological divide specifically impacting black nursing students negatively (Hassan & Daniel 2020). This is supported by Early and Hernandez (2021) in that COVID-19 pandemic has exacerbated the digital divide and perpetuated systemic racism and poverty. Additionally, Risling (2017) is of the view that the nurses of 2025 and beyond will most certainly inhabit a very different practice environment than what exists today, and technology will be key in this transformation, thus requiring the nurse educators to prepare now to lead these practitioners into the future. The question is, how can the nurse educator do this when we are still faced with social justice issues such as the digital divide in nursing education?

In conclusion, nurse educators and researchers are urged to investigate ways of bridging the digital divide, eliminating social and educational disparities among the larger population of nursing students who come from families living below the poverty line. We cannot talk social justice and equity when students are hungry, have no educational technology devices and have no connectivity or electricity for that matter.
